# Development and validation of a novel competing risk model for predicting survival of esophagogastric junction adenocarcinoma: a SEER population-based study and external validation

**DOI:** 10.1186/s12876-021-01618-7

**Published:** 2021-01-26

**Authors:** Tongbo Wang, Yan Wu, Hong Zhou, Chaorui Wu, Xiaojie Zhang, Yingtai Chen, Dongbing Zhao

**Affiliations:** 1 Department of Pancreatic and Gastric Surgical Oncology, National Cancer Center/National Clinical Research Center for Cancer/Cancer Hospital, Chinese Academy of Medical Sciences and Peking Union Medical College, No. 17 PanjiayuanNanli, Chaoyang District, Beijing, 100021 China; 2 Key Laboratory of Carcinogenesis and Translational Research (Ministry of Education), Department of Pathology, Peking University Cancer Hospital & Institute, Beijing, 100142 China

**Keywords:** Esophagogastric junction adenocarcinoma, Nomogram, Competing risk analysis, SEER database

## Abstract

**Background:**

Adenocarcinoma in Esophagogastric Junction (AEG) is a severe gastrointestinal malignancy with a unique clinicopathological feature. Hence, we aimed to develop a competing risk nomogram for predicting survival for AEG patients and compared it with new 8th traditional tumor-node-metastasis (TNM) staging system.

**Methods:**

Based on data from the Surveillance, Epidemiology, and End Results (SEER) database of AEG patients between 2004 and 2010, we used univariate and multivariate analysis to filter clinical factors and then built a competing risk nomogram to predict AEG cause-specific survival. We then measured the clinical accuracy by comparing them to the 8th TNM stage with a Receiver Operating Characteristic (ROC) curve, Brier score, and Decision Curve Analysis (DCA). External validation was performed in 273 patients from China National Cancer Center.

**Results:**

A total of 1755 patients were included in this study. The nomogram was based on five variables: Number of examined lymph nodes, grade, invasion, metastatic LNs, and age. The results of the nomogram was greater than traditional TNM staging with ROC curve (1-year AUC: 0.747 vs. 0.641, 3-year AUC: 0.761 vs. 0.679, 5-year AUC: 0.759 vs. 0.682, 7-year AUC: 0.749 vs. 0.673, *P* < 0.001), Brier score (3-year: 0.198 vs. 0.217, *P* = 0.012; 5-year: 0.198 vs. 0.216, *P* = 0.008; 7-year: 0.199 vs. 0.215, *P* = 0.014) and DCA. In external validation, the nomogram also showed better diagnostic value than traditional TNM staging and great prediction accuracy.

**Conclusion:**

We developed and validated a novel nomogram and risk stratification system integrating clinicopathological characteristics for AEG patients. The model showed superior prediction ability for AEG patients than traditional TNM classification.

## Background

Despite the incidence trend continuously decreased over the past few decades, gastric cancer remains the fifth most common malignant tumor and ranks third in cancer-related mortality in the world [1, 2]. An growing number of population-based studies had observed that the incidence of adenocarcinoma in the esophagogastric junction (AEG) presenting a significantly rising tendency [3–5]. According to the latest 8th edition of the American Joint Committee on Cancer (AJCC) Cancer Staging Manual, the Tumor-Node-Metastasis (TNM) staging system of AEG had been divided due to its unique clinicopathological characteristicsl: viz. tumors with their epicenter within the proximal 2 cm of the esophagogastric junction (EGJ) invaded (Siewert I/II) are classified as the version of TNM-esophagus cancer, and tumors with their epicenter more than 2 cm distal from the EGJ would be classified as TNM-gastric cancer [6]. However, this staging strategy for AEG only focused on the ‘location’ of invasion, neglecting other critical clinical features, such as age, sex and the number of resected lymph node (LN), which could be predicting factors that influencing patients’ prognosis [7–10]. Thus, the prognostic evaluation system for AEG needs to be further explored.

In general, the survival of cancer patients may be affected by more than two events, and only one event occurs finally [11]. Those events other than the one of interest are called competing risk events. The traditional survival analysis may overestimate the cumulative incidence by treating competing events as censored events, which could be improved by the competing risk analysis. Nomogram, a simple graphical linear prediction model, is widely used for cancer prognosis [12]. Hence, in this study, we aimed to explore a new classification system by competing risk model through the population-based Surveillance, Epidemiology and End Results (SEER) database and further develop and externally validate a nomogram for predicting survival for AEG patients.

## Methods

### Training cohort and data acquisition

Patient data were obtained from the SEER website (http://seer.cancer.gov/) using SEER*stat version 8.3.5. In total, data from 2004 to 2010 of 11,639 esophagogastric junction (EGJ) cancer patients over 18 years old were initially analyzed. The inclusion criteria were as followed: (1) patients with histological confirmed adenocarcinomas in EGJ; (2) patients who received surgery and complete pathological information can be achieved; (3) without distant metastasis; The exclusion criteria were as followed: (1) patients with multiple primary tumors; (2) primary EGJ cancers with other histology; (3) primary EGJ adenocarcinomas without histological confirmation; (4) patients with distant metastasis; (5) patients without complete pathological information; (6) patients with follow-up time less than 3 months. Finally, we extracted clinicopathological variables of 1755 patients including age, gender, race, location of the tumor, TNM staging, the grade of the tumor, histological grade, number of examined LNs, number of positive LNs, tumor size, and survival months.

### External validation cohort and data

To further validate our new predicting model, we sought an external validation cohort from patients diagnosed from October 2006 to December 2018 and underwent radical resection in China National Cancer Center. The validation cohort included 273 AEG patients who were recruited according to inclusion and exclusion criteria same as the training cohort. The time of last follow-up was March 2019. All study procedures were approved by the Institutional Review Board at the China National Cancer Center.

### Exploration of a new evaluation system and presentation of nomograms

We regarded AEG cause-specific death and other causes of death as two competing events in our competing-risk analysis. The multivariate proportional sub-distribution hazard model was used to calculate the adjusted sub-distribution hazard ratio (SHR) of the new examined evaluation system. Variables associated with AEG cause-specific death with a *P* value of < 0.1 in the univariate analysis, or a *P* value of < 0.05 in the initial multivariate analysis, were included as variables in the final multivariate analysis. We not only built the proportional sub-distribution hazard model to predict cause-specific death for patients, but also competing-risk nomograms based on Fine and Gray’s model [13]. For comparing the predicted probability with points observed at a certain time, a calibration plot was used. If both the predicted and observed probabilities in any given pair lie on the 45° line, it implies that both probabilities match well to each other and the model is ideal. The discrimination of the model was assessed by areas under receiver operating characteristic curves (AUC) [14]. If the AUC > 0.8, it indicates that the discriminatory accuracy of a model is good. The discrimination and calibration of the model were also measured by the Brier score at the same time [15]. The decision curve analysis (DCA) was then used to estimate the clinical usefulness and net benefit of the predictive models, as well as compare them with the traditional TNM staging system of the training cohort [16, 17]. This method was capable to visualizes the clinical consequences of a treatment strategy at each threshold probability.

### Statistical analysis

All statistical analyses were performed using R software, version 3.3.3 (Institute for Statistics and Mathematics, Vienna, Austria; http://www.r-project.org). Statistical significance was set at two-sided *P* < 0.05.

## Results

### Patients characteristics

**Fig. 1:**
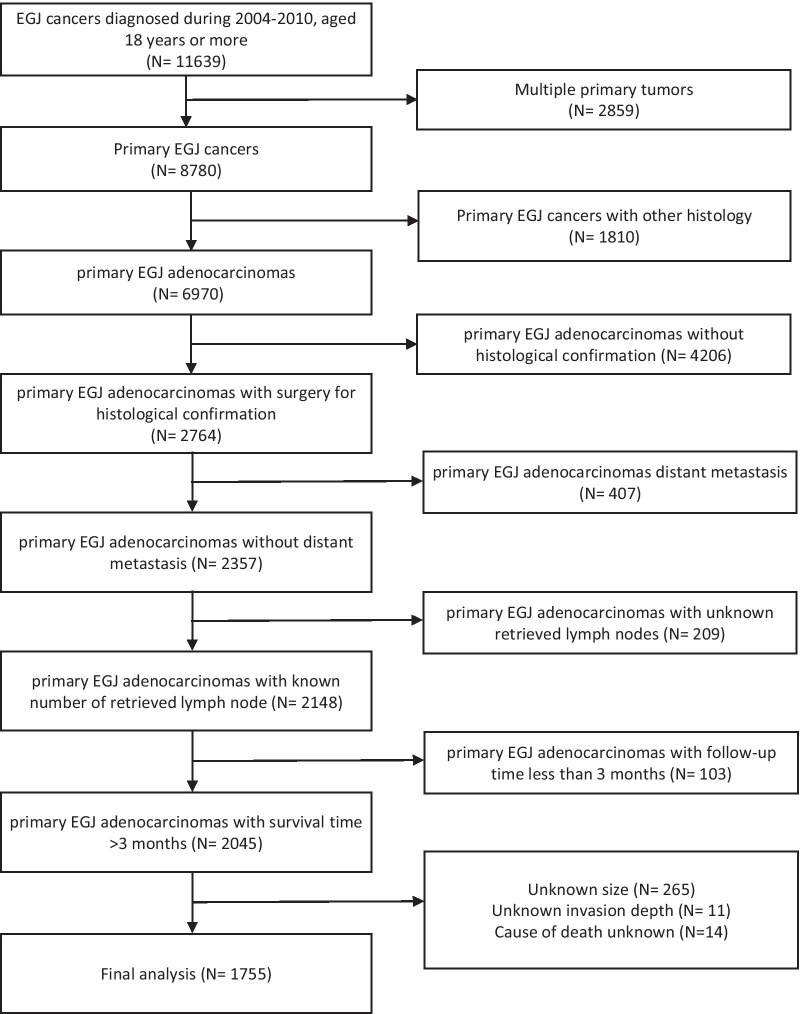
Flowchart of the training cohort patients selection for this study

In total, 11,639 adults were diagnosed with EGJ cancer from 2004 to 2010, 2859 of which were excluded due to having multiple primary tumors. Furthermore, patients with other histology (N = 1810) except adenocarcinoma, without histological confirmation (N = 4206), with distant metastasis (N = 407), or with unknown examined LNs (N = 209) were also excluded. In addition, primary AEG with a follow-up of less than 3 months (N = 103), unknown size (N = 265), unknown invasion depth (N = 11), or cause of unknown death (N = 14) were excluded as well. In the end, 1755 cases were included in further analysis (Fig. 1), comprising 373 females (21.2%) and 1382 males (78.8%), with sixty years being used as a cut-off for elderly people. The T stage ranged from T1 to T4 (N = 355, 227, 768, 405, respectively), and the N stage from N0 to N3 (N = 716, 391, 340, 308, respectively). The external validation cohort comprised 73 females (26.7%) and 200 males (73.3%), with 81.6% low grade and 18.4% high grade differentiation. The details of the baseline characteristics of participants of two cohorts are shown in Table 1.

**Table 1:** Characteristics of two cohorts of patients with AEG

Characteristics	Training cohort (N = 1755)		Validation cohort (N = 273)	
	No. of patients	%	No. of patients	%
Race				
White	1523	86.8	0	0
Black	81	4.6	0	0
Others	151	8.6	273	100
Sex				
Female	373	21.2	73	26.7
Male	1382	78.8	200	73.3
Age (year)				
< 60	674	38.4	154	56.4
≥ 60	1081	61.6	119	43.6
Grade				
Low grade	946	53.9	223	81.6
High grade	809	46.1	50	18.4
T stage				
T1	355	20.2	47	17.2
T2	227	12.9	38	13.9
T3	768	43.8	63	23.1
T4	405	23.1	125	45.8
N stage				
N0	716	40.8	25	9.2
N1	391	22.2	46	16.8
N2	340	19.4	68	24.9
N3	308	17.6	134	49.1
Size (cm)				
< 1.0	83	4.7	8	2.9
< 2.0	189	10.8	42	15.4
< 3.0	265	15.1	49	17.9
< 5.0	584	33.3	102	37.4
≥ 5.0	634	36.1	72	26.4
Number of examined lymph nodes				
< 5	128	7.3	3	1.1
< 10	323	18.4	5	1.8
< 15	428	24.4	20	7.3
≥ 15	876	49.9	245	89.7
Follow-up time (months, median, IQR)	33 (14,79)		21 (12–40)	

*IQR* interquartile range

### AEG survival prediction model

In the final multivariate proportional sub-distribution hazard model of clinical characteristics for the prognosis of AEG in the training cohort, an age of > 60 years (SHR 1.389, *P* < 0.001), high T stage (T1-4, SHR 1.592, 2.167, 2.555, respectively, *P* < 0.001), high N stage (N0-3, SHR 1.814, 2.505, 3.335, *P* < 0.001), or high Grade (SHR 1.281, *P* < 0.001) was associated with a poor prognosis, while the number of examined LNs presented to be a protective factor (< 10, < 15, ≥ 15, SHR 0.751, 0.635, 0.540, *P* < 0.001). The results of multivariate analysis are listed in detail in Table 2.

**Table 2:** Multivariate analysis for exploring potential risk factors for prognosis of AEG

Characteristics	Initial multivariate model		Final multivariate model		
	SHR (95% CI)	*P*	beta	SHR (95% CI)	*P*
Age (year)					
< 60	Reference			Reference	
≥ 60	1.393 (1.223–1.588)	< 0.001	0.329	1.389 (1.220–1.583)	< 0.001
T stage					
T1	Reference			Reference	
T2	1.604 (1.204 -2.137)	< 0.001	0.465	1.592 (1.211–2.093)	< 0.001
T3	2.207 (1.698–2.869)	< 0.001	0.773	2.167 (1.710–2.747)	< 0.001
T4	2.606 (1.990–3.411)	< 0.001	0.938	2.555 (2.000–3.265)	< 0.001
N stage					
N0	Reference			Reference	
N1	1.831 (1.525–2.198)	< 0.001	0.596	1.814 (1.515–2.172)	< 0.001
N2	2.536 (2.089–3.077)	< 0.001	0.918	2.505 (2.073–3.028)	< 0.001
N3	3.417 (2.750–4.247)	< 0.001	1.205	3.335 (2.702–4.116)	< 0.001
Grade					
Low grade	Reference			Reference	
High grade	1.277 (1.120–1.457)	< 0.001	0.248	1.281 (1.124–1.460)	< 0.001
Number of examined lymph nodes					
< 5	Reference			Reference	
< 10	0.736 (0.555–0.975)	0.032	− 0.286	0.751 (0.569–0.993)	0.040
< 15	0.630 (0.477–0.832)	0.001	− 0.455	0.635 (0.481–0.838)	0.013
≥ 15	0.535 (0.410–0.698)	< 0.001	− 0.616	0.540 (0.414–0.704)	< 0.001
Race					
White	Reference				
Black	0.973 (0.713–1.329)	0.860			
Others	0.829 (0.662–1.039)	0.100			
Sex					
Female	Reference				
Male	0.965 (0.825–1.129)	0.660			
Size (cm)					
< 1.0	Reference				
< 2.0	1.179 (0.732–1.899)	0.500			
< 3.0	1.087 (0.682–1.731)	0.730			
< 5.0	1.121 (0.707–1.777)	0.630			
≥ 5.0	1.048 (0.659–1.668)	0.840			

*SHR* sub-distribution hazard ratio, *CI* confidential interval

### Construction of the competing risk nomogram

**Fig. 2:**
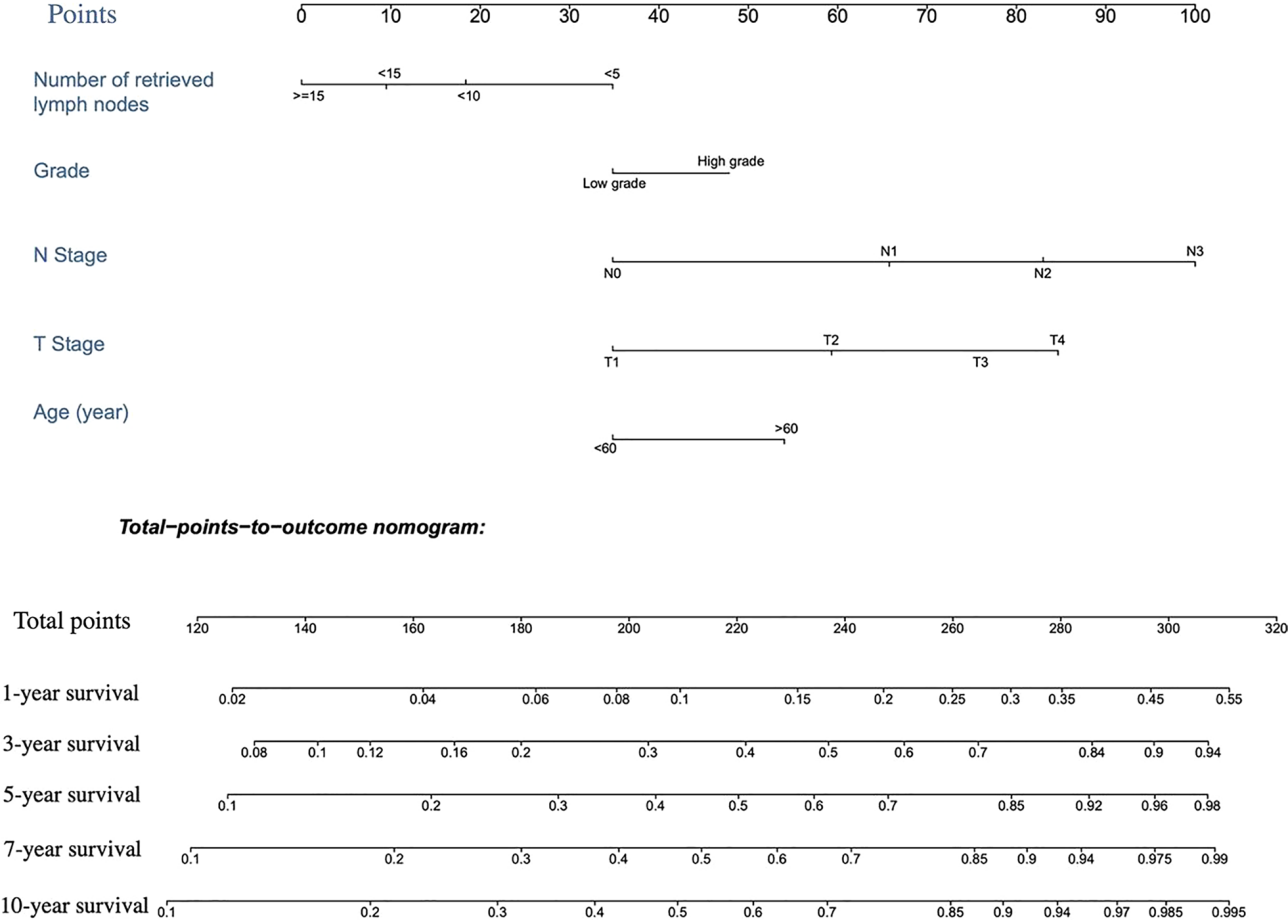
Nomogram predicted 1- to 10-year cancer specific death for patients with resected AEG using five available clinical characteristics. To use the nomogram, an individual patient’s value is located on each variable axis, and a line is drawn upward to determine the number of points received for each variable value. The sum of these numbers is located on the Total Points axis, and a line is drawn downward to the survival axes to determine the likelihood of 1- to 10-year survival

The AEG cause-specific death predicting model of the nomogram was established based on a selection of prognostic factors (Fig. 2). The nomogram showed the N stage to be the most impactful factor of prognosis, followed by the T stage, and then age, with the amount of examined LNs and grade having only a modest effect on survival. Each subtype of the variables was assigned a score. A straight line to determine the estimated probability of survival can be drawn at each time point on the total point scale, according to the total point.

### Evaluation and Validation of the nomogram

**Fig. 3:**
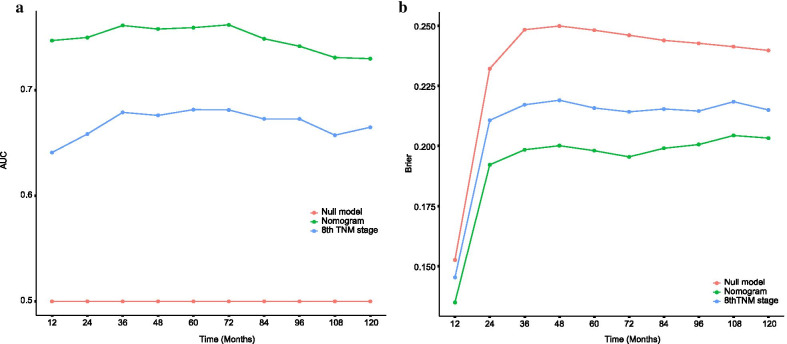
AUC (**a**) and Brier score (**b**) of the Nomogram and 8th TNM Stage in prediction of prognosis of patients from 1-year to 10-year point. AUC: areas under the receiver operating characteristic curve

**Fig. 4:**
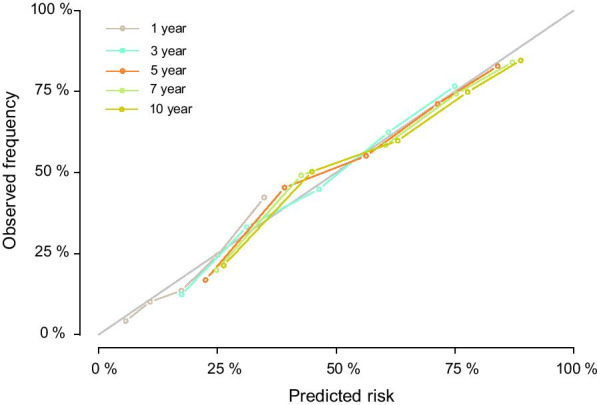
The calibration curves for predicting patient survival of the training cohort at 1-, 3-, 5-year, 7-year, 10-year point. Nomogram-predicted cancer specific survival is plotted on the x-axis; actual cancer specific survival is plotted on the y-axis. A plot along the 45-degree line would indicate a perfect calibration model in which the predicted probabilities are identical to the actual outcomes

In the analysis of specificity, we used both the receiver operating characteristic (ROC) curve and Brier score to evaluate the diagnostic value and accuracy of the nomogram model. With respect to the ROC curve, the nomogram model was greater than traditional TNM staging in the training cohort (1-year AUC: 0.747 vs. 0.641, 3-year AUC: 0.761 vs. 0.679, 5-year AUC: 0.759 vs. 0.682, 7-year AUC: 0.749 vs. 0.673, respectively, *P* < 0.001, Fig. 3). The Brier score is a measure of overall performance and captures aspects of both calibration and discrimination. It is a representation of the difference between the predicted probability and the actual outcome. The score ranges from 0 to 1, with values closer to 0 indicating better predictive ability. In terms of the Brier score, the accuracy of the nomogram was also better than traditional TNM stage at 3-year point (0.198 vs. 0.217, *P* = 0.012), 5-year point (0.198 vs. 0.216, *P* = 0.008), and 7-year point (0.199 vs. 0.215, *P* = 0.014) (Fig. 3). The calibration curves showed the dots close to a 45° diagonal line, indicating that the nomogram were well calibrated (Fig. 4).

**Fig. 5:**
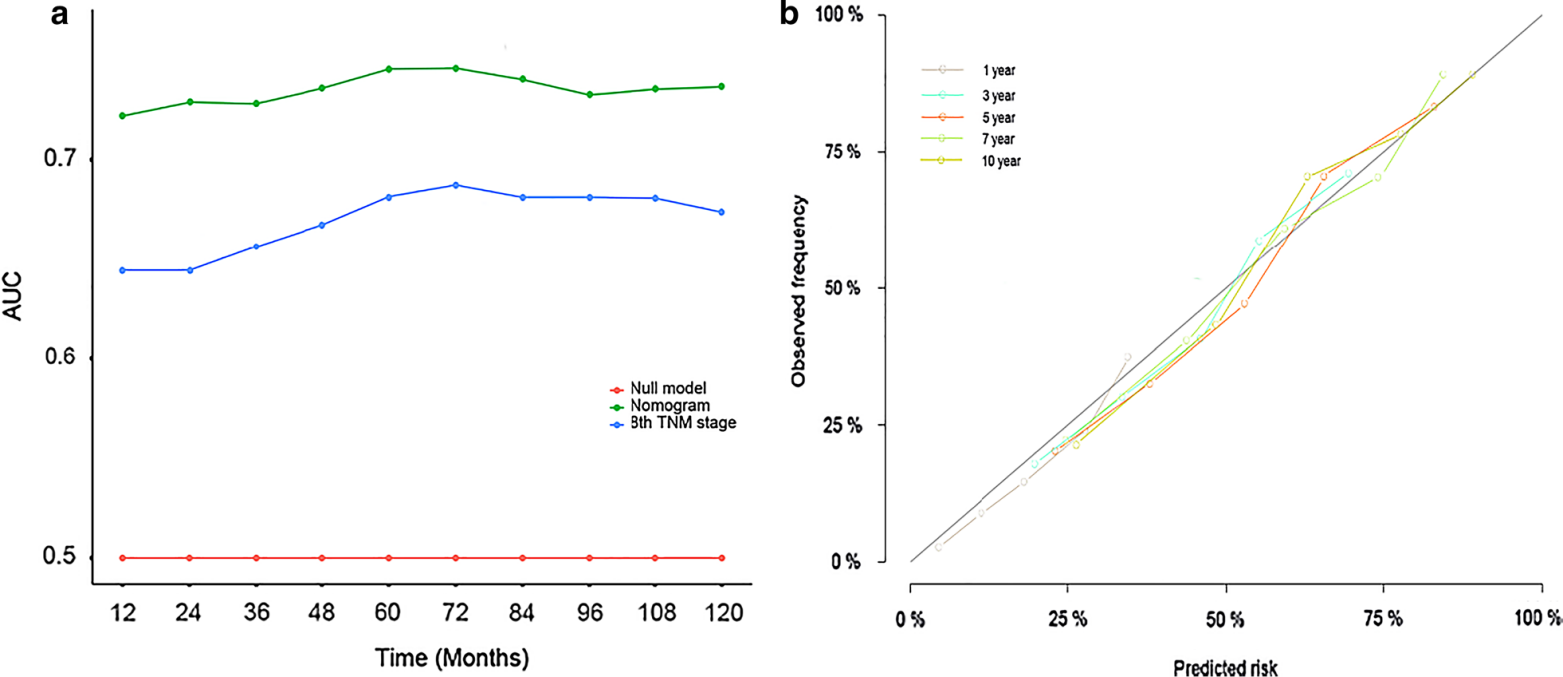
**a** AUC of the Nomogram and 8th TNM Stage in prediction of prognosis of the validation cohort patients from 1-year to 10-year point. **b** The calibration curves for predicting patient survival of the validation cohort at 1-, 3-, 5-year, 7-year, 10-year point

To further externally validate the nomogram, we used ROC curves and calibration curves to evaluate the prediction accuracy of the new model. The ROC curves presented a better diagnostic value than traditional TNM staging and the calibration curves presented an acceptable consistency between the model prediction and the actual observation for 1-, 3-, 5-, 7- and 10-year point (Fig. 5).

**Fig. 6:**
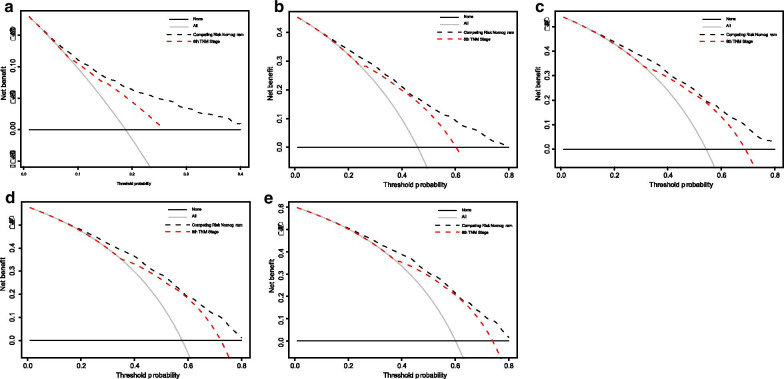
Decision curve analysis for the Competing Risk Nomogram and the 8th TNM Stage in prediction of prognosis of patients at 1, 3, 5, 7, 10-year point

We then used DCA to compare the clinical usefulness of the nomogram and traditional TNM staging. By decision curve analysis, the nomogram was better than traditional TNM staging in clinical conditions (Fig. 6). Compared with traditional TNM staging, the nomogram showed a larger net benefit across the range of death risk in the analysis.

## Discussion

As a junctional tumor type between the esophagus and stomach, the definition, evaluation, and management of AEG remains elusive. Based on the 8th AJCC TNM stage classification, AEG patients could receive better evaluation and management [18]. However, the new complex classification may sometimes confuse clinicians, resulting in unfavorable evaluation and therapy [6]. Ergo, a new specific evaluation and classification system for AEG is urgently needed. In this study, a new classification system was developed through the use of a competing risk model nomogram for predicting survival in patients with AEG. This nomogram is based on five variables: number of LNs examined, grade, invasion (T stage), metastatic LNs (N stage), and age. This nomogram produced more accurate predictions on the survival of patients than the pathologic 8th TNM classification and showed better clinical usefulness throughout the time during which patients were analyzed as assessed by DCA.

Due to the unique anatomic location, the AJCC classification of AEG remains a topic of debate in recent years. An accurate staging system is required for clinicians to choose the best follow-up treatment [19]. In the 8th edition of AJCC classification, more attention was paid to changes and developments leading to better clinical decision making and predictive accuracy. Separated staging of AEG reflects the individualized approach taken by AJCC. In the new AJCC staging system, a more complex classification separated by 3 different groups was introduced, namely clinical (cTNM), pathologic (pTNM), and post-neoadjuvant pathologic (ypTNM) stage groups [18]. The clinical stage is defined using physical examination, endoscopy, and imaging examination, which shows big heterogeneity between different surgeons. In clinical practice, pathologic stage groups showed the most widely distributed survival [20, 21]. In addition, a direct comparison of the different editions AJCC classification is possible only for pathologic staging. Therefore, we used the pathologic stage groups of the 8^th^ TNM staging method in our comparison.

Traditional TNM classification stratified the AEG into 3 grouping methods: the pathologic depth, number of metastatic LNs, and distant metastasis of the tumor. The survival data are usually accompanied by multiple outcomes, which may have a competitive association [10, 22, 23], resulting in overestimation of the cumulative incidence. A nomogram is a well-analyzed statistic tool which provides a comprehensive probability of outcome [12]. A prior study comparing nomogram with 7^th^ AJCC classification, which included six clinical associated factors (age, sex, depth of invasion, metastasized LNs, examined LNs, histological grade), showed greater accuracy for the TNM classification [8]. In clinical practice, however, we found the outcome of AEG could be blocked by many other events [5, 24]. Therefore, we used a new competing risk nomogram to reduce the influence of these outcomes [25, 26]. In our study, we used the number of examined LNs, grade, N stage, T stage, and age as the classification factors, part of which is in consensus with the TNM stage system. This simple nomogram could eliminate possible influence from other lethal factors and be useful for clinicians in practice.

In our nomogram, the number of examined LNs was considered as another variable of evaluating the survival in addition to normal factors in the AJCC staging system. The number of examined LNs presented to be a protective factor (< 10, < 15, ≥ 15, SHR 0.751, 0.635, 0.540, *P* < 0.001) in this nomogram, indicating that resection of more LNs leads to a better survival. Several trials also recommended the number of examined LNs as a great predicting factor in the staging system of AEG [27–29]. Different surgical methods may determine the number of LN examinations [30]. The choice of surgery depends on the type of AEG: with type I being treated as esophageal cancer, and type II and III regarded as gastric cancer [10, 30]. More examined LNs may represent a more exhaustive surgical dissection and less residing positive LNs. Moreover, not only could the dissection procedure of the surgeon affect the number of examined LNs, but so could the LN searching of the pathologist. Thus, we could mark the examined LNs as an evaluation for pathological credibility.

This work also has some limitations. Firstly, as cancer biology and validation of biologic factors has evolved, it has become more effective in predicting the outcome of cancer [18, 23]. The 8^th^ AJCC classification recommended some of these factors, with strong evidence showing great accuracy in AEG. Due to the missing data from the SEER database, we were unable to make a full comparison to the whole 8^th^ classification; further study combining biologic data may lead to a more ‘personalized’ approach. Secondly, the SEER database is based on retrospective data collection, with diagnosis and surgery all depending on different doctors from several different medical centers. Moreover, the current work was also limited by the inability to involve some recognized prognostic factors such as Siewert type, surgical operation, radiation and chemotherapy due to lack of detailed information of therapy related variables in the SEER database. In addition, the missing data during collection caused many patients to be excluded, which might lead to skewed results.

## Conclusion

We developed and validated a novel nomogram and risk stratification system integrating clinicopathological characteristics for AEG patients. The model showed superior prediction ability for AEG patients than traditional TNM classification.

## Data Availability

The datasets generated and analyzed during the current study are available in the SEER database (https://seer.cancer.gov/) and from the corresponding authors upon reasonable request.

## Abbreviations

AEG: Adenocarcinoma in the esophagogastric junction
TNM: Tumor-Node-Metastasis staging classification
AJCC: The American Joint Committee on Cancer
EGJ: Esophagogastric junction
LN: Lymph node
SEER: Surveillance, Epidemiology and End Results
SHR: Sub-distribution hazard ratio
AUC: Areas under receiver operating characteristic curves
DCA: Decision curve analysis
ROC: Receiver operating characteristic

## References

[1] Ferlay J, Parkin DM, Steliarova-Foucher E (2010). Estimates of cancer incidence and mortality in Europe in 2008. Eur J Cancer.

[2] Ferlay J, Soerjomataram I, Dikshit R, Eser S, Mathers C, Rebelo M, Parkin DM, Forman D, Bray F (2015). Cancer incidence and mortality worldwide: sources, methods and major patterns in GLOBOCAN 2012. Int J Cancer.

[3] Blot WJ, Devesa SS, Kneller RW, Fraumeni JF (1991). Rising incidence of adenocarcinoma of the esophagus and gastric cardia. JAMA.

[4] Kusano C, Gotoda T, Khor CJ, Katai H, Kato H, Taniguchi H, Shimoda T (2008). Changing trends in the proportion of adenocarcinoma of the esophagogastric junction in a large tertiary referral center in Japan. J Gastroenterol Hepatol.

[5] Devesa SS, Blot WJ, Fraumeni JF (1998). Changing patterns in the incidence of esophageal and gastric carcinoma in the United States. Cancer.

[6] Rice TW, Gress DM, Patil DT, Hofstetter WL, Kelsen DP, Blackstone EH (2017). Cancer of the esophagus and esophagogastric junction-Major changes in the American Joint Committee on Cancer eighth edition cancer staging manual. CA Cancer J Clin.

[7] Suh YS, Lee KG, Oh SY, Kong SH, Lee HJ, Kim WH, Yang HK (2017). Recurrence pattern and lymph node metastasis of adenocarcinoma at the Esophagogastric junction. Ann Surg Oncol.

[8] Zhou Z, Zhang H, Xu Z, Li W, Dang C, Song Y (2015). Nomogram predicted survival of patients with adenocarcinoma of esophagogastric junction. World J Surg Oncol.

[9] Lagarde SM, ten Kate FJ, Reitsma JB, Busch OR, van Lanschot JJ (2006). Prognostic factors in adenocarcinoma of the esophagus or gastroesophageal junction. J Clin Oncol.

[10] Hasegawa S, Yoshikawa T (2010). Adenocarcinoma of the esophagogastric junction: incidence, characteristics, and treatment strategies. Gastric Cancer.

[11] Dignam JJ, Zhang Q, Kocherginsky M (2012). The use and interpretation of competing risks regression models. Clin Cancer Res.

[12] Iasonos A, Schrag D, Raj GV, Panageas KS (2008). How to build and interpret a nomogram for cancer prognosis. J Clin Oncol.

[13] Fine JP, Gray RJ (1999). A proportional hazards model for the subdistribution of a competing risk. Publ Am Stat Assoc.

[14] Hanley JA, McNeil BJ (1983). A method of comparing the areas under receiver operating characteristic curves derived from the same cases. Radiology.

[15] Callegaro D, Miceli R, Bonvalot S, Ferguson P, Strauss DC, Levy A, Griffin A, Hayes AJ, Stacchiotti S, Pechoux CL (2016). Development and external validation of two nomograms to predict overall survival and occurrence of distant metastases in adults after surgical resection of localised soft-tissue sarcomas of the extremities: a retrospective analysis. Lancet Oncol.

[16] Blanche P, Dartigues JF, Jacqmin-Gadda H (2013). Estimating and comparing time-dependent areas under receiver operating characteristic curves for censored event times with competing risks. Stat Med.

[17] Wu S, Chen JN, Zhang QW, Tang CT, Zhang XT, Tang MY, Li XB, Ge ZZ (2018). A New metastatic lymph node classification-based survival predicting model in patients with small bowel adenocarcinoma: a derivation and validation study. EBioMedicine.

[18] Amin MB, Greene FL, Edge SB, Compton CC, Gershenwald JE, Brookland RK, Meyer L, Gress DM, Byrd DR, Winchester DP (2017). The Eighth Edition AJCC Cancer Staging Manual: Continuing to build a bridge from a population-based to a more "personalized" approach to cancer staging. CA Cancer J Clin.

[19] Sobin LH (2003). TNM: evolution and relation to other prognostic factors. Semin Surg Oncol.

[20] Rice TW, Rusch VW, Apperson-Hansen C, Allen MS, Chen LQ, Hunter JG, Kesler KA, Law S, Lerut TE, Reed CE (2009). Worldwide esophageal cancer collaboration. Dis Esophagus.

[21] Rice TW, Ishwaran H, Blackstone EH, Hofstetter WL, Kelsen DP, Apperson-Hansen C (2016). Worldwide Esophageal Cancer Collaboration I: Recommendations for clinical staging (cTNM) of cancer of the esophagus and esophagogastric junction for the 8th edition AJCC/UICC staging manuals. Dis Esophagus.

[22] Ielpo B, Pernaute AS, Elia S, Buonomo OC, Valladares LD, Aguirre EP, Petrella G, Garcia AT (2010). Impact of number and site of lymph node invasion on survival of adenocarcinoma of esophagogastric junction. Interact Cardiovasc Thorac Surg.

[23] Siewert JR, Feith M, Stein HJ. Biologic and clinical variations of adenocarcinoma at the esophago-gastric junction: relevance of a topographic-anatomic subclassification. J Surg Oncol 2005, 90(3):139–146; discussion 146. 10.1002/jso.20218 15895452

[24] Siegel RL, Miller KD, Jemal A (2018). Cancer statistics, 2018. CA Cancer J Clin.

[25] Li J, Li X, Gu J, Ma X, Xue F (2018). A competing-risks nomogram for predicting probability of death from CRC in Chinese Han patients with Stage I-III CRC. Jpn J Clin Oncol.

[26] van Vugt JLA, Alferink LJM, Buettner S, Gaspersz MP, Bot D, Darwish Murad S, Feshtali S, van Ooijen PMA, Polak WG, Porte RJ (2018). A model including sarcopenia surpasses the MELD score in predicting waiting list mortality in cirrhotic liver transplant candidates: A competing risk analysis in a national cohort. J Hepatol.

[27] Kang CH, Kim YT, Jeon SH, Sung SW, Kim JH (2007). Lymphadenectomy extent is closely related to long-term survival in esophageal cancer. Eur J Cardiothorac Surg.

[28] Boshier PR, Anderson O, Hanna GB (2011). Transthoracic versus transhiatal esophagectomy for the treatment of esophagogastric cancer: a meta-analysis. Ann Surg.

[29] Lagarde SM, Reitsma JB, Ten Kate FJ, Busch OR, Obertop H, Zwinderman AH, Moons J, van Lanschot JJ, Lerut T (2008). Predicting individual survival after potentially curative esophagectomy for adenocarcinoma of the esophagus or gastroesophageal junction. Ann Surg.

[30] Liu K, Feng F, Chen XZ, Zhou XY, Zhang JY, Chen XL, Zhang WH, Yang K, Zhang B, Zhang HW (2019). Comparison between gastric and esophageal classification system among adenocarcinomas of esophagogastric junction according to AJCC 8th edition: a retrospective observational study from two high-volume institutions in China. Gastric Cancer.

